# Targeted kinase inhibition relieves slowness and tremor in a *Drosophila* model of LRRK2 Parkinson’s disease

**DOI:** 10.1038/s41531-017-0036-y

**Published:** 2017-12-04

**Authors:** Amy C. Cording, Nicolas Shiaelis, Stavroula Petridi, C. Adam Middleton, Laurence G. Wilson, Christopher J. H. Elliott

**Affiliations:** 1 Department of Biology, University of York, York, YO1 5DD UK; 2 Department of Physics, University of York, York, YO1 5DD UK

## Abstract

In a number of *Drosophila* models of genetic Parkinson’s disease (PD) flies climb more slowly than wild-type controls. However, this assay does not distinguish effects of PD-related genes on gravity sensation, “arousal”, central pattern generation of leg movements, or muscle. To address this problem, we have developed an assay for the fly proboscis extension response (PER). This is attractive because the PER has a simple, well-identified reflex neural circuit, in which sucrose sensing neurons activate a pair of “command interneurons”, and thence motoneurons whose activity contracts the proboscis muscle. This circuit is modulated by a single dopaminergic neuron (TH-VUM). We find that expressing either the *G2019S* or *I2020T* (but not *R1441C*, or kinase dead) forms of human *LRRK2* in dopaminergic neurons reduces the percentage of flies that initially respond to sucrose stimulation. This is rescued fully by feeding l-DOPA and partially by feeding kinase inhibitors, targeted to LRRK2 (LRRK2-IN-1 and BMPPB-32). High-speed video shows that *G2019S* expression in dopaminergic neurons slows the speed of proboscis extension, makes its duration more variable, and increases the tremor. Testing subsets of dopaminergic neurons suggests that the single TH-VUM neuron is likely most important in this phenotype. We conclude the *Drosophila* PER provides an excellent model of *LRRK2* motor deficits showing bradykinesia, akinesia, hypokinesia, and increased tremor, with the possibility to localize changes in neural signaling.

## Disease models: A reflex reaction

A simple reflex in flies can be used to test the effectiveness of therapies that slow neurodegeneration in Parkinson’s disease (PD). Christopher Elliott and colleagues at the University of York in the United Kingdom investigated the contraction of the proboscis muscle which mediates a taste behavior response and is regulated by a single dopaminergic neuron. Flies bearing particular mutations in the PD-associated gene leucine-rich repeat kinase 2 (LRRK2) in dopaminergic neurons lost their ability to feed on a sweet solution. This was due to the movement of the proboscis muscle becoming slower and stiffer, hallmark features of PD. The authors rescued the impaired reflex reaction by feeding the flies l-DOPA or LRRK2 inhibitors. These findings highlight the proboscis extension response as a useful tool to identify other PD-associated mutations and test potential therapeutic compounds.

## Introduction

Parkinson’s disease (PD) is a progressive neurodegenerative condition characterized by pathological loss of dopaminergic neurons in the *substantia nigra*. The reduction in dopamine in the projections to the striatum leads to a range of motor disorders, characterized by bradykinesia (slower movements), hypokinesia (a reduction in the amplitude of movements), akinesia (absence of movement altogether), and tremor.

Although the cause of the majority of PD is unknown, a small proportion is inherited (see, for a review, ref. ^1^) with the most common genetic form being due to mutations in *LRRK2* (*Leucine-Rich Repeat Kinase 2*).

This gene is translated into a large, multi-domain protein, and the pathogenic mutations include *G2019S* and *I2020T,* ^2^ in which the kinase activity is increased,^3^ and *R1441C*, a mutation in which the GTPase activity is thought to be reduced.^4^

The excellent genetic toolkit provided by *Drosophila* led to the creation of fly models of PD. These reflect many features of the disease (loss of dopaminergic neurons, reduced movement, mitochondrial abnormalities, oxidative stress. and visual deficits).^5,6^ Many labs have adopted the fly negative geotaxis assay (sometimes called the “startle response assay”) as their measure of movement.^7–9^ In this, the speed at which flies walk up a glass cylinder in response to a sharp tap is recorded. Although PD-mimic flies have reduced movement, it is hard to specify exactly where the changes are taking place (response to the startle stimulus or gravity, or effects on the central pattern generator or motor neurons or changes directly affecting the leg muscles themselves). This assay also fails to discriminate between the different possible movement defects (akinesia, hypokinesia, and bradykinesia). We suggest the requirement for another, simpler assay system.

This is reinforced by the difficulty of determining which of the ~125 dopaminergic neurons in the fly CNS^10,11^ are important in the negative geotaxis response. Although dopaminergic innervation of the mushroom body by 15 “PAM” neurons plays a major role in this negative geotaxis response,^12^ the subsequent neuronal pathway is unclear. Further, manipulations of PD-related transgenes often lead to the loss of a relatively small proportion of dopaminergic neurons, with many clusters remaining unaffected. For example, with the *LRRK2-G2019S* mutation, the protocerebral posterior medial (PPM) cluster dropped from 14 to 12 dopaminergic neurons but the protocerebral anterior lateral (PAL) cluster remained unaffected.^13^ Throughout the literature, the multiple processes involved in slowed negative geotaxis combined with the observed small loss of dopaminergic neurons act to obscure the functional relationship. To progress, we need to link a precise measurement of movement with the physiology of a few specific dopaminergic neurons.

An exciting solution to this problem is provided by the discovery that a single dopaminergic neuron strongly modulates the fly proboscis extension response (PER).^14^

**Fig. 1:**
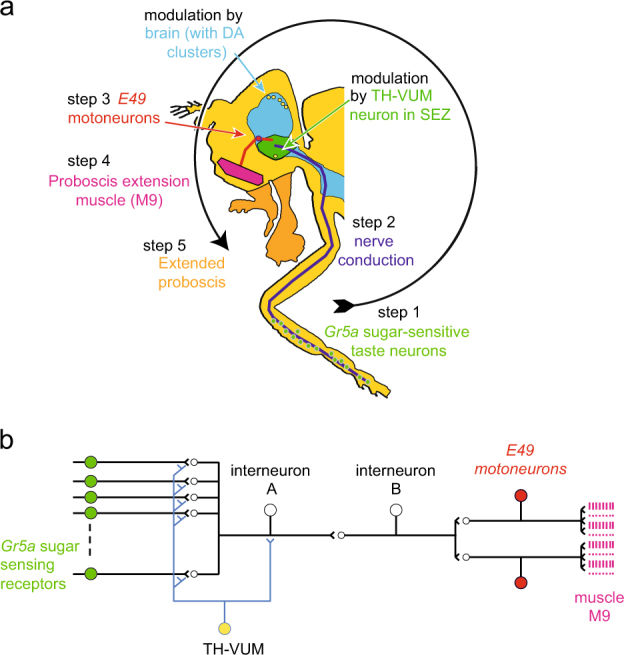
The proboscis extension response (PER) of *Drosophila*. **a** The PER takes place when sugar-sensitive (*Gr5a*) neurons on the legs respond (step 1) and signal to the sub-esophageal zone of the CNS (SEZ, step 2). This leads to activation of the *E49* motoneurons for the proboscis extension muscle (step 3), muscle contraction (step 4), and extension of the proboscis (step 5). In the sub-esophageal zone, the neuronal signal is modulated by a dopaminergic neuron, TH-VUM, and by other inputs from CNS neurons, possibly including other dopaminergic neurons. **b** Schematic neural circuit, showing the modulation of the sensory neuron–interneuron–motoneuron axis by TH-VUM. Other interneurons also modulate the proboscis extension response, as reviewed recently.^17^ **a** Modified after refs. ^52,53^; **b** after refs. ^14,15,19^

As a fly walks into a solution containing sucrose, the *Gr5a* chemosensory cells on its front legs are activated (Fig. 1, step 1). Their axons project to the sub-esophageal zone of the CNS (SEZ; the part signaling the taste response; Fig. 1, step 2). Within the SEZ, the chemosensory inputs activate the interneuronal pathway,^15^ leading to the pharyngeal *E49* motoneurons, whose action potentials elicit contraction of the M9 muscle (Fig. 1, step 3). This well characterized neuronal pathway results in the reflex extrusion of the proboscis towards the food (Fig. 1, step 4), allowing the fly to ingest the solution. Although the sensory and motor steps in this pathway have been well defined (see for a review ref. ^16^ or ref. ^17^), the interneuronal steps mostly remain to be described.

One well-defined neuron that modulates the PER is TH-VUM, a single, unpaired neuron in the SEZ, which makes output synapses onto the sense cells and interneurons^14,18,19^ (Fig. 1). Strong activity in the TH-VUM leads to contraction of the proboscis muscle, blocking the output of the TH-VUM reduces the probability of a sucrose-induced PER. The frequency of action potentials in the TH-VUM correlates with the length of starvation.^14^ Interestingly, the TH-VUM fires steadily in a way reminiscent of mammalian *substantia nigra* dopaminergic neurons.^20^

We have now found that expression of *LRRK2-G2019S*, the most common cause of genetic PD, in dopamine neurons results in a reduced PER, with bradykinesia, akinesia, and tremor, and that this is rescued by l-DOPA or by kinase inhibitors targeted at LRRK2.

## Results

### Upregulation of LRRK2 kinase activity in dopaminergic neurons causes akinesia

**Fig. 2:**
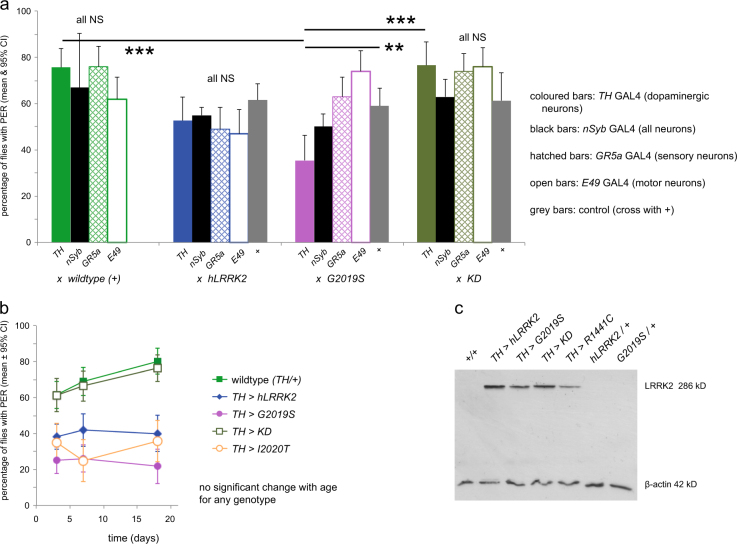
The PER shows bradykinesia with dopaminergic expression of two *LRRK2* mutations (*G2019S*, *I2020T*) that have increased kinase activity. This reduces the proportion of flies that respond to sucrose stimulation with a proboscis extension response. **a** Comparison of the expression of a Parkinson’s mutant with upregulated kinase activity (*LRRK2-G2019S*) with wild-type *hLRRK2*, and a kinase dead line (*KD, LRRK2-G2019S-K1906M*). Each group of bars shows the effect of transgene expression in dopaminergic neurons (using the *TH*-GAL4), the sugar-sensitive neurons on the legs (*Gr5a*-GAL4), in the proboscis motoneurons (*E49*-GAL4) in relation to outcross controls (+) in which no transgene was expressed. *N* = 1972, at least 60 flies per sample. **b** Dopaminergic expression of two increased kinase lines (*G2019S, I2020T*) reduces the PER at all ages. There is no decline in the proportion of flies showing PER with age, up to 28 days. *N* = 1839, at least 75 flies in each sample. **c** Western blot showing that dopaminergic expression of *hLRRK2* or *KD* leads to stronger staining than *LRRK2-G2019S*, while the *R1441C* is slightly weaker. In **a** and **b**, wild-type (+) is *TH*/*w* ^*a*^; in **c**, *w* ^−^ and *CS*. Data derived from different flies in **a** and **b**. In **c**, flies were raised together, so that the samples derive from the same experiment and were processed in parallel

In order to test the neuronal specificity of the PD-related mutation *LRRK2-G2019S*, we first expressed this in each of the components of the PER reflex pathway, recording the proportion of a population of starved flies that extended their proboscis in response to a moderate (100 mM) sugar stimulus (Fig. 2a, b). Strikingly, when we expressed *LRRK2-G2019S* in the dopaminergic neurons with *TH*-GAL4 (*tyrosine hydoxylase* GAL4), the proportion of young flies responding was about half that of control genotypes (no transgene expressed, *χ* ^2^-post-hoc test, *p* < 0.001; from 76–35%; Fig. 2a). The same result was seen in a second sample, where the proportion of *TH>G2019S* flies responding was also less than half that of the wild type (Fig. 2b). In contrast, there was no effect of *G2019S* when driven in all neurons (*nSyb*-GAL4, *p* = 0.17), or in just the sensory neurons (*Gr5a*-GAL4, *p* = 0.11), or solely in proboscis motoneurons (*E49-*GAL4, *p* = 0.21).

In order to establish if this was specific for the *G2019S* mutation, we also tested a second PD-related mutation in which kinase activity is increased (*I2020T*), a mutation in the GTPase domain (*R1441C*), a kinase dead form (*LRRK2-G2019S-K1906M*), and the normal human form (*hLRRK2*). Of these, only dopaminergic expression of *I2020T* had any specific effect, reducing the proportion of flies that responded from 61 to 35 % (*χ* ^2^-post-hoc test, *p* < 0.001; Fig. 2b). We did note a general reduction on all flies containing the *hLRRK2* (Fig. 2a, b), due perhaps to the increased expression levels of this gene (Fig. 2c). In turn, the lack of effect of *TH>R1441C* might be due to the weaker expression (Fig. 2c), based on our hypothesis that the impact of any one LRRK2 mutant on PER is correlated with the level of LRRK2 protein production in the dopaminergic neurons.

We tested a second set of independently generated *LRRK2* transgenic flies,^21^ and found the same: only 36% of *TH>G2019S* flies responded compared with 52% of *TH>hLRRK2* and 61% of *TH/+* (*χ* ^2^-post-hoc test, *G2019S* vs *hLRRK2*, *p* = 0.04; *G2019S* vs wild type, *p* = 0.0034).

To determine if the PER response was age dependent, we tested flies from 3 days to over 18 days, and found none of the genotypes showed any age dependent change (Fig. 2b). Dopaminergic expression of *G2019S* or *I2020T* reduced the proportion of flies responding to the sucrose stimulus at all ages.

We found no effect of genotype on mass at 20 days (non-transgene control vs *TH>hLRRK2* vs *TH>G2019S*, F_2,26 df_ = 0.003, *p* = 0.99), suggesting the reduced frequency of PER seen in starved flies was not preventing them from increasing their mass when provided with food ad libitum. However, by 28 days, only 57% of flies were alive, with increased mortality in each of the *TH>hLRRK2*, *TH>G2019S*, and *TH>I2020T* compared to the *TH/+* control (*χ* ^2^-post-hoc test, *p* < 0.01 for each; survival 38, 53, 57, 80%, respectively).

These experiments show that the expression of high kinase forms of *LRRK2* reduces the proportion flies showing PER, i.e. they show akinesia.

### Rescue of akinesia by l-DOPA and kinase inhibitors

**Fig. 3:**
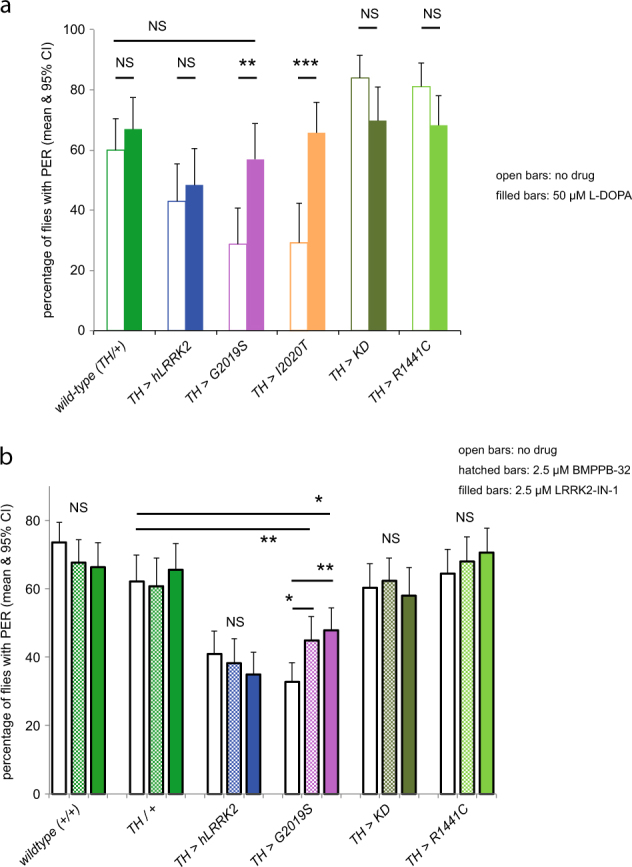
l-DOPA and LRRK2-specific kinase inhibitors both rescue the bradykinesia induced by kinase mutations in the PER. **a** Feeding 50 μM l-DOPA rescues the reduction in PER by dopaminergic expression of *LRRK2-G2019S* or *LRRK2-I2020T* to wild-type levels. l-DOPA has no effect on *hLRRK2*, kinase dead (*KD*, *LRRK2-G2019S-K1906M*) or the GTPase mutant (*R1441C*). All transgenes expressed by the *TH*-GAL4. The wild type (+) is *w* ^−^. *N* = 902, at least 60 flies per sample. **b** LRRK2 kinase inhibitors rescue the reduction in PER caused by dopaminergic expression of *LRRK2-G2019S*. Flies were fed with either 2.5 μM BMPPB-32 or LRRK2-IN-1. Neither drug affects the controls or flies with dopaminergic expression of *hLRRK2*, *KD*, or *R1441C*. Exact genotypes: +/+ *CS/w¯*; *TH/+ TH* GAL4*/CS. N* = 3387, at least 130 flies per sample

Since the standard symptomatic treatment for PD is l-DOPA, we tested if this compound would rescue the PER deficit induced by dopaminergic expression of *G2019S* or *I2020T*. In both cases, the administration of l-DOPA to the adult flies raised the proportion of flies that respond to sucrose to control levels (Fig. 3a). This was not due to an increase in general responsiveness, because we saw no effect of l-DOPA on the flies expressing *hLRRK2*, *KD*, or *R1441C*.

As the most common form of genetic PD is caused by the *LRRK2-G2019S* mutation, with its increased kinase activity, a promising therapy would be to deploy kinase inhibitors targeted at LRRK2. The first of these to be developed was LRRK2-IN-1.^22^ Application of 2.5 μM LRRK2-IN-1 partially rescued the frequency of PER responses, from 32 to 47% (*p* = 0.002, Fig. 3b). Since LRRK2-IN-1 has off-target effects, in vitro^23,24^ and in vivo,^6^ we next tested the more specific compound BMPPB-32, which also gave a partial rescue, to 44% (Fig. 3b). The proportion of flies showing the PER was the same for LRRK2 and BMPPB-32 (*p* = 0.077) and both were below the wild-type level (73%).

We conclude that drugs targeted at LRRK2 ameliorate the reduced PER response of *TH>G2019S* flies.

### Dopaminergic expression of LRRK2-G2019S slows movement and increases tremor in the PER

**Fig. 4:**
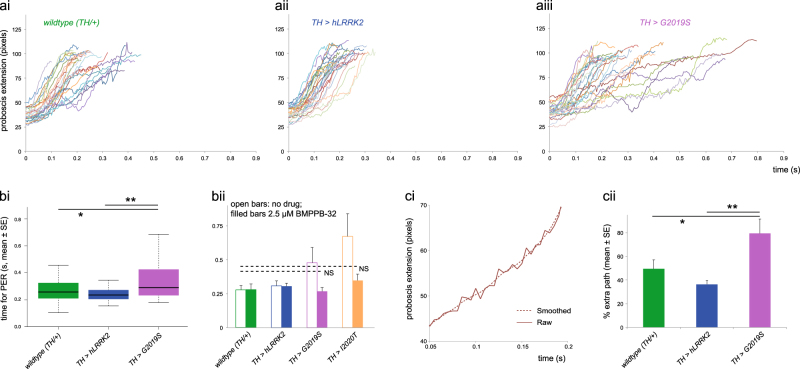
Dopaminergic expression of *LRRK2-G2019S* (*TH* GAL4) slows and increases tremor in the PER. **a** The raw plot of the distance between the eye and the tip of the proboscis shows that the PER takes longer, and is more variable, with *TH>G2019S* than with *TH*>*hLRRK2* or the control with no transgene expressed (*TH/+*). **b** Summary showing the longer (and more variable) time taken by the PER in *TH>G2019S* and *TH>I2020T* flies. Feeding 2.5 μM BMPPB-32 reverts the time taken to control levels. **c** Fitting a piecewise cubic spline to each trace (i) generates a smooth curve, allowing the calculation of the extra path taken by the proboscis. The mean extra path (ii) is longer for *TH>G2019S* than the wild type or *TH>hLRRK2*, indicating increased tremor. Data for **a**, **b**i, and **c** from the same data set, *N* = 80, at least 26 in each group; for **b**ii *N* = 141, at least 15 in each group. *TH/+* is *TH/w¯*

For *TH>G2019S* flies showing a PER, recordings were made using a camera with high frame rate (200 per second), and digitized the distance from the eye to the end of the proboscis, to measure the movement during the PER. Individual traces showed that some *TH>G2019S* flies had very slow PER, taking three or even four times longer than the median wild-type control (Fig. 4a), with the increase in standard error being very noticeable (Fig. 4b). A second mutant, *I2020T*, also showed a slower PER (Fig. 4bii). For both *TH>G2019S* and *TH>I2020T* flies, the speed of the PER is fully rescued to wild type by feeding 2.5 μM BMPPB-32.

Using the independently generated *G2019S* and *hLRRK2* lines,^21^ we also found a slower PER with *TH>G2019S*. In this case the duration of the PER increased from 0.30 ± 0.025 s (*TH/+*) to 0.57 ± 0.11 s (*TH>G2019S*) (mean ± SE, Tukey-post-hoc test, *p* = 0.017). The *TH>hLRRK2* was the same as *TH/+* (*p* = 0.81).

Additionally, it appeared that the *TH>G2019S* traces were more irregular. To assess this quantitatively, each trace was fitted by a smooth curve (a piecewise cubic spline), and the deviation of the actual trace from smoothed determined (Fig. 4ci). This showed that the *TH>G2019S* flies had a much longer “path”, about twice that of the control flies. The proboscis does not move out in a smooth trajectory, but oscillates, showing tremor. No such changes were seen in the *TH>hLRRK2* flies; they were the same as the no-transgene cross (Fig. 4cii).

Thus dopaminergic expression of *LRRK2-G2019S* induces bradykinesia and tremor as well as akinesia.

### The single dopamine neuron (TH-VUM) is mainly responsible for akinesia

Our next step was to test which dopaminergic neurons are responsible for akinesia in the PER response, focusing on the difference between flies with increased kinase activity (*G2019S* or *I2020T*) and the kinase dead (*KD*) line.

**Fig. 5:**
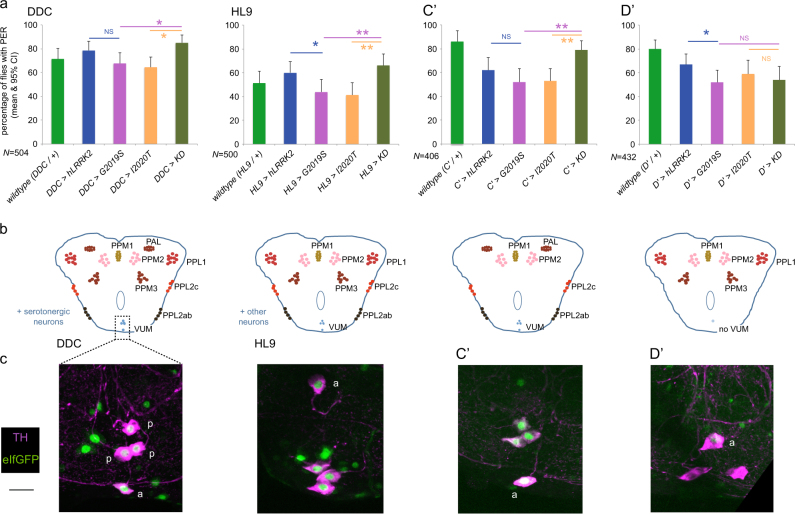
The presence of dopaminergic TH-VUM neuron is essential for the *G2019S*/*I2020T*-mediated reduction in PER. **a** Proportion of flies responding when *LRRK2* transgenes are expressed in different subsets of the dopaminergic neurons, using the *DDC, HL9, C′* or *D′* GAL4 drivers. There is no difference between the increased kinase mutants (*G2019S*/*I2020T*) and the kinase-dead construct (*KD, G2019S-K1906M*) with the *D′* GAL4, which does not express in the TH-VUM neurons. All the other GAL4 lines tested express in the TH-VUM neurons and show a smaller response in *G2019S*/*I2020T* than in *KD*. Exact genotypes: + is *w* ^*a*^. **b** Summary maps of the expression patterns of the GAL4 drivers used in **a**. Figures redrawn after Mao and Davis (2009).^10^ **c** The lack of TH-VUM in the D′ line is confirmed anatomically. Each panel shows the projection of a confocal stack through the sub-esophageal zone (as marked in the first panel by the dotted box). Neurons marked by expression of *eIfGFP* under the control of the relevant GAL4 and stained by anti-TH antibody. The SEZ contains a single anterior cell (“a”) and a group of three posterior cells (“p”), marked with anti-TH antibody With *DDC, HL9*, and C′ GAL4 drivers, all four SEZ neurons were GFP positive. With D′, the nucleus of the anterior neuron “a” has a weak GFP signal, but the three posterior neurons marked with anti-TH antibody do not fluoresce green (note the cytoplasm of the two left posterior cells is merged in this projection of the z-stack, but their empty nuclei are still visible). Scalebar 20 μm

There are eight clusters of dopaminergic neurons in the fly CNS (Fig. 5b), and a range of GAL4 drivers have been developed to target various subsets of these. We started with a GAL4 driver *DDC,* ^25^ which has been widely used for studies of negative geotaxis and which gives generalized dopaminergic neuron expression, as well as expressing in serotonergic neurons. As with the *TH*-GAL4 (Fig. 1), fewer flies expressing *G2019S* or *I2020T* with *DDC* showed the PER compared with those flies expressing *LRRK2*-*KD* (Fig. 5). We next tested *HL9* (ref. ^26^) which expresses in all the dopaminergic neurons except for the PAL cluster (Fig. 5b), though it may only label a proportion of each cluster. It may also label a few non-dopaminergic neurons (Fig. 5c). Again, the *G2019S* and *I2020T* flies were less responsive than the *KD* flies. With the *C*′-GAL4 line,^27^ which expresses in all dopaminergic neurons except the PPM3 and PPL1 clusters, we saw the same pattern: *G2019S* and *I2020T* flies showed less PER than the *KD* flies. All these GAL4 lines express in the VUM dopaminergic neurons. The final GAL4 tested, *D′* ^27^ expresses in all dopaminergic neurons except the PAL, PPL2 and does not express strongly in the VUM neurons. Remarkably, with the *D′-*GAL4, flies expressing *G2019S*, *I2020T*, and *KD* forms of *LRRK2* all showed an equal response to sucrose, strikingly different from all the other dopaminergic GAL4s.

We next used a nuclear GFP (eIfGFP; Fig. 5c) to confirm the expression pattern of the GAL4 lines in the SEZ. *DDC, HL9*, and *C′*, all showed a group of three GFP-positive neurons located posteriorly plus a fourth cell anteriorly. One of the three posterior cells is the unpaired midline TH-VUM and the other two are descending neurons (left and right DA-DNs), whose activity represents leg movements.^28^ The *D′>eIfGFP* fluorescence pattern is noticeably different: only the anterior cell shows any GFP signal (and that weakly), but the three posterior neurons not being labeled at all. The presence of the three posterior neurons is confirmed by the anti-TH staining.

Consequently, we suggest that the effect of expressing *LRRK2-G2019S* in dopaminergic neurons is mostly mediated by the single TH-VUM neuron, because the two descending neurons do not show a direct link to feeding behavior. Only when *G2019S* is expressed in the TH-VUM do we see the reduction in PER, i.e. akinesia. However, we cannot rule out an effect of the PPL2 neurons, which also modulate the feeding system^29^ as they are also not targeted by the *D′*-GAL4.

## Discussion

Our principle finding is that expressing *LRRK2* forms with increased kinase activity (*G2019S*, *I2020T*) in sets of dopaminergic neurons that include the TH-VUM is sufficient to induce akinesia, bradykinesia, and tremor in the fly PER. Although previous work with flies and rodents have identified movement disorders in PD models, our PER assay uniquely identifies the components of the response, in the context of changes mostly due to to a single dopaminergic neuron (TH-VUM).

A key point is that our PER assay demonstrates dopaminergic-bradykinesia even at 3 days. In comparison, data from negative geotaxis (startle-induced climbing response) assays of *G2019S, I2020T*, or *R1441C* transgenics is more complex, depending on age and genotype. One report shows that while locomotion in *DDC>I2020T* flies is already compromised at 5 days,^30^ another study shows that *TH>I2020T* flies show little deficit at any age.^31^ This may be because *DDC*-GAL4 includes serotonergic, as well as dopaminergic neurons, and thus more cells compared with *TH-*GAL4. Using *DDC*-GAL4, to express *LRRK2* in old (>5 weeks) flies, *G2019S* and *R1441C* movement is shown to be reduced,^32,33^ while younger flies show no deficit. In contrast, our data show movement deficits in young *TH>G2019S* and *TH>I2020T* flies, which are maintained over the first few weeks of adult life. Indeed, LRRK2 mutations already start to affect *Drosophila* larvae, indicating effects at an earlier timepoint.^21,34,35^

One disadvantage of working with older flies is that, by 5 weeks, a proportion of flies will have died, potentially those most strongly influenced by the transgene, so that negative geotaxis assays may underestimate the real impact of LRRK2 mutations. Our assay has the advantage of working at 3 days, before flies have started to die, and potentially could be developed so that the same individual fly might be tested at different time points. This would permit comparison of the individual and population responses.

In our visual assays with *TH>G2019S*, we found that 3-day-old flies showed no detectable visual deficits, though younger flies (1-day-old) had overactive vision, and old flies (28-day-old, or visually stressed) had much reduced response.^6,36,37^ Overactivity has also been reported in young transgenic LRRK2 rats, followed later by loss of movement.^38–40^ We have not tested the feeding response of flies less than 3 days old, because these newly emerged flies rest and expand their cuticle, and are not feeding: this makes them unsuitable for PER assay.

However, the PER of our mildly starved 3-day-old *TH>G2019S* flies is already reduced, and remains well below wild-type levels for at least 18 days. A more pronounced PER deficit might arise in older flies (5 weeks), and/or those kept at 29 °C to enhance transgene expression.

Further, the movement deficits in our PER assay on flies starved for 2–3 h are mainly a consequence of expressing *G2019S* in a single dopaminergic neuron, TH-VUM, rather than the mixed effect of a range of dopaminergic clusters.

In this respect the PER assay differs from both negative geotaxis and our visual assay (three different kinds of dopaminergic neuron are present in the retina). However, we note that longer term modulation of feeding appears to involve other dopaminergic clusters, interacting in the mushroom bodies.^41^ Additionally, our PER deficit occurs in young flies in which the TH-VUM is still present, offering the potential to understand the processes by which LRRK2 leads to neuronal silencing in a single identified neuron.

We find that both the akinesia and bradykinesia components of the *TH>G2019S* effect on PER are dependent on the kinase role of LRRK2. We observe no effect of expressing the *KD* (kinase-dead, *G2019S-K1906M*) form of *LRRK2*, although the expression level is stronger than *G2019S*. In this respect, it resembles the visual assay, where expressing *G2019S*, but not this *KD* construct led to retinal neurodegeneration.^37^ The *TH>R1441C* flies also showed no reduction in PER, or in visual degeneration^37^ though it is possible that this is because *R1441C* is not so effectively expressed. The rescue by the specific LRRK2 inhibitor, BMPPB-32, argues that the reduction in PER is a consequence of phosphorylation of substrate(s) by LRRK2. We previously showed this inhibitor was effective in a visual assay, reverting *TH>G2019S* phenotypes in both young and old flies.^6,36^ Another specific inhibitor LDN-73794 prevents loss of *DDC>G2019S* induced locomotion in old flies.^42^ The PER assay has an advantage over the climbing assay (startle response), where degeneration is usually measured at 4–5 weeks, as our flies only need to be fed with the inhibitors for 3 days, reducing compound requirements.

Genetically activating or silencing the TH-VUM respectively increase or decrease the probability of a PER.^14^ Thus, our data showing kinase active *LRRK2* transgenes in the TH-VUM reduce PER could be explained by a reduction in dopamine release by this neuron. We hypothesize that expressing *G2019S* in the TH-VUM could lead to either (i) failure of TH-VUM neurites to grow, (ii) a reduction in its tonic firing, (iii) less dopamine synthesis, or (iv) lower probability of release of dopamine onto the reflex pathway. Cultured mammalian neurons, fly motoneurons, and sensory cells all have reduced neurites with *G2019S.* ^23,43,44^ While it is possible that *G2019S* also reduces neuritic branching in the TH-VUM neuron, our data rather favor hypotheses (iii) or (iv) since we found that feeding flies l-DOPA rescued the *TH>G2019S* loss of PER. Reduced dopamine levels have been reported with *DDC>G2019S*, and with ubiquitous expression of an increased kinase form of the fly homolog *dLRRK.* ^45,46^ In both *Drosophila* and mammals, l-DOPA can cross the blood–brain barrier, but dopamine cannot.^47^ Thus we suggest that uptake of l-DOPA into the TH-VUM leads to increased dopamine levels and release, rescuing the effect of *TH>G2019S*. Increasing the amount of dopamine released onto the sugar-sensing *Gr5a* neurons would then rescue the proportion of flies that show the PER (akinesia), while release onto second order, local interneurons, might affect the motoneurons and thence speed (bradykinesia), and tremor of the proboscis extension. Such a dual output onto *Gr5a* neurons and onto local interneurons is suggested by the fact that 2.5 μM BMPPB-32 fully rescues bradykinesia, but only partially rescues akinesia. Although a number of interneurons in the SEZ with roles controlling proboscis extension, ingestion, and memory have recently been identified (e.g. see refs. ^48–50^), the link between *Gr5a* sense cells and the *E49* motoneurons remains to be established.

## Methods

Flies, *Drosophila melanogaster*, were raised at 25 °C on cornmeal-sugar-agar-yeast food. The following GAL4 lines were used: *TH (tyrosine hydoxylase)* GAL4,^51^ *DDC*-GAL4,^25^ *HL9*,^26^ or the *C′* and *D′*-GAL4 stocks,^27^ the pan-neuronal *nSyb*-GAL4 (Stephen Goodwin), the sensory *Gr5a-*GAL4 and motoneuron *E49-*GAL4.^18^ The UAS lines were: wild-type *hLRRK2* or *LRRK2-G2019S,* ^32^ *hLRRK2*-*I2020T* and the kinase dead line *LRRK2-G2019S*-*K1906M* (hereafter, *KD*),^21^ and *hLRRK2*-*R1441C*;^31^ *eIfGFP* (*eIF4AIII::GFP*, Andreas Prokop). In some confirmatory experiments, independent *LRRK2-G2019S* and *hLRRK2* lines were used.^21^ The lab stocks of *CS* (*Canton-S*), *w* ^*1118*^ (*w*¯), and *w* ^*apricot*^ (*w* ^*a*^ Bloomington stock 148) provided “wild-type” outcross controls.

PER was performed (A.C.C.) by collecting male flies of known age at the start of the working day, under CO_2_ anesthesia, and sticking them ventral side up to card with rubber cement (Fixo Gum). Flies were left to recover for 2–3 h at 25 °C. They were presented with a droplet of 100 mM sucrose solution to the legs, and the immediate PER/no PER scored (response in <2 s). Experiments were designed so that each graph plotted here comes from flies scored over three adjacent days, with the genotypes mixed each day, to allow for the small variations in food and environmental conditions. Power calculations indicate that a “medium” effect size, with a sample of 500 flies, and 16 df, would be detected at the 1 % level >98% of the time.

Drugs were fed to adult flies from eclosion until testing. l-DOPA (Sigma) was added to food (final concentration 50 μM). BMPPB-32 and LRRK2-IN-1 (Lundbeck) were dissolved to give a final concentration in the food at 2.5 μM.

PER was filmed using a Mikrotron MC-1362 camera mounted on a Zeiss Stemi microscope. Videos were acquired at 200 frames/s; sample movies for a wild type and *TH>G2019S* flies are presented in Movies S1 and S2. Only the first PER of each fly was analyzed. In Matlab, the eye and tip of the proboscis were marked and their separation was determined for each frame individually. The analyses of Movies S1 and S2 are shown in Movies S3 and S4, respectively.

Western blots were performed as described^6^ using the heads of 3-day-old female flies, raised at 29 °C using anti-LRRK2 (Neuromab, clone N241A/34, 1:1000) and anti-β-actin (Proteintech, 1:180,000, loading control). The data are representative of three blots.

Immunocytochemistry was as described recently,^37^ using mouse anti-TH (Immunostar, 1:1000) and driving *eIfGFP* using the required GAL4 line. All data are from male flies, aged 3–5 days. No anti-GFP was used in the data chosen for illustration. The brightness and contrast of the images was adjusted in ImageJ so that the cells could be seen in both color channels, as each GAL4 drove GFP with a different intensity in the VUM neurons. Original images available on request to cje2@york.ac.uk Representative data from at least three preparations are shown.

### Statistics

For analysis of the proportion of flies showing a PER, statistical significance was determined using the *χ* ^2^-post-hoc test in the “Fifer” package of R. Confidence limits were determined using the Binomial test in R. Measurements of the speed of the PER were analyzed by ANOVA and Tukey post-hoc tests. For a “medium” effect size, with 26 flies in each of three samples, and the probability of 0.05, the power is 63%. *N* values for each genotype/treatment are included in Supplementary Table 1.

### Data availability statement

The data that support the findings of this study are available from the corresponding author upon reasonable request.

## Electronic supplementary material

Supplementary Table 1

Movie S1

Movie S2

Movie S3

Movie S4
